# Vascular smooth muscle cell differentiation-2010

**DOI:** 10.1016/S1674-8301(10)60026-7

**Published:** 2010-05

**Authors:** Joseph M. Miano

**Affiliations:** Aab Cardiovascular Research Institute, University of Rochester School of Medicine & Dentistry, Rochester, New York 14642, USA

## Abstract

Vascular smooth muscle cells have attracted considerable interest as a model for a flexible program of gene expression. This cell type arises throughout the embryo body plan via poorly understood signaling cascades that direct the expression of transcription factors and microRNAs which, in turn, orchestrate the activation of contractile genes collectively defining this cell lineage. The discovery of myocardin and its close association with serum response factor has represented a major break-through for the molecular understanding of vascular smooth muscle cell differentiation. Retinoids have been shown to improve the outcome of vessel wall remodeling following injury and have provided further insights into the molecular circuitry that defines the vascular smooth muscle cell phenotype. This review summarizes the progress to date in each of these areas of vascular smooth muscle cell biology.

Cell lineage determination and differentiation are essential for the normal development of the human body. Cellular identity is hard-wired in an array of signaling pathways that converge upon the nuclear genome to orchestrate proper patterns of gene expression requisite for the specific functions performed by the ∼250 distinct cell types. Thus any given cell type's identity and phenotypic characteristics are determined primarily by the signaling input, both intrinsic and extrinsic, and the subset of nuclear genes that are transcriptionally and post-transcriptionally controlled through such signaling events. One cell type of paramount importance in the developing and postnatal body plan is the vascular smooth muscle cell (VSMC). These cells are derived from a variety of distinct regions in the developing embryo[1] and function as structurally supportive cells lying immediately subjacent to the inner lining endothelial cells of blood vessels. VSMC also control the caliber of blood vessels (especially resistance vessels in the microcirculation) and thus the flow of blood through their intrinsic contractile properties. The purpose of this review is three-fold. First, the molecular definition of VSMC is reviewed with a brief listing of the major signaling pathways involved in the specification of this cell lineage. Second, the major transcriptional switch for VSMC lineage differentiation will be described. Finally, the role of retinoids as ligands for nuclear receptors in VSMC will be outlined with special attention to retinoid-response genes that function to maintain a normal VSMC differentiated phenotype. The reader should consult additional, complementary reviews on the subject of vascular smooth muscle cell differentiation[2]–[5].

## DEFINING VASCULAR SMC

The positioning of VSMC within the tunica media of blood vessels offers a histological definition of this cell type. However, like all human cell types, VSMC may also be defined in molecular terms by the expression of a subset of the > 23,000 genes in the human genome. Over the last 25 years, major progress has been made in defining the unique transcriptome of VSMC[3],[6]. Molecular cloning studies and careful developmental expression assays have revealed a unique molecular signature for VSMC that collectively define this cell lineage in molecular terms (***Table 1***). As a muscle type, many of the genes expressed in VSMC encode for elements of the contractile apparatus. For example, the smooth muscle isoforms of myosin heavy chain (*MYH11*), alpha actin (*ACTA2*), gamma actin (*ACTG2*), and calponin (*CNN1*) as well as SM22 alpha (*TAGLN*) and smoothelins (*SMTNA* and *SMTNB*) all show adult VSMC-specific patterns of expression. Interestingly, however, many of these genes show transient expression in developing skeletal and/or cardiac muscle[7]–[11]. The mechanism for such early embryonic expression across muscle types is unknown but probably relates to a combination of shared expression of key transcription factors as well as the absence of silencing pathways that would normally repress VSMC-specific genes in sarcomeric muscle. A larger question is why VSMC-specific genes are even expressed in skeletal and/or cardiac muscle during development; there have been no studies, to date, examining the physiological role of VSMC-specific protein activity in embryonic skeletal or cardiac muscle. As discussed in section two below, the major transcriptional switch for VSMC differentiation controls virtually all of the aforementioned genes.

**Table 1:** Gene symbols (and aliases) defining the molecular signature of VSMC

Gene name	Function
MYH11 (smooth muscle myosin heavy chain)	Contractile
MYLK_v7 (telokin)	Contractile
ACTA2 (smooth muscle alpha actin)	Contractile
CNN1 (smooth muscle calponin)	Contractile
TPM1 (alpha tropomyosin)	Contractile
TPM2 (beta tropomyosin)	Contractile
CALD_v1 (heavy caldesmon)	Contractile
MYLK_v6 (smooth muscle myosin light chain kinase)	Contractile
ACTN1 (alpha actinin)	Contractile
ACTG2 (smooth muscle gamma actin)	Cytoskeleton
SMTNA (smoothelin A)	Cytoskeleton
SMTNB (smoothelin B)	Cytoskeleton
VCL_v1 (meta-vinculin)	Cytoskeleton
DMD (dystrophin)	Cytoskeleton
TAGLN (Sm22 alpha)	Cytoskeleton
APEG1 (SPEG complex locus)	Cytoskeleton
DES (desmin)	Cytoskeleton
ITGA8 (alpha 8 integrin)	Cytoskeleton
ITGA1 (alpha 1 integrin)	Cytoskeleton
HEY2 (CHF-1)	Transcription
BARX2b	Transcription
MYOCD (myocardin)	Transcription
SRF (serum response factor)	Transcription
AEBP1 (ACLP)	Transcription
HDAC8 (histone deacetylase 8)	Transcription
PTK2 (FRNK)	Signaling
NOTCH3	Signaling
ELN (elastin)	Structural
LPP (lipoma preferred partner)	Protein binding
GLMN (glomulin)	Protein binding
CSRP1 (CRP1)	Protein binding
PGM5 (phosphoglucomutase)	Metabolism
HRC (histine-rich calcium binding protein)	Calcium binding
KCNMB1 (maxi-K beta 1 subunit)	Ion channel

The majority of genes in the human genome undergo alternative splicing[12]. In this manner, VSMC contractile genes (*e.g., MYH11*) can undergo alternative splicing to generate functionally distinct proteins. Such splicing events likely explain how such a complex organism as a human being is endowed with only a marginal increase in gene number over more simple animals (*e.g., Caenorhabditis elegans* with only 19,000 genes). Some VSMC-specific genes arise from alternative splicing of more widely expressed genes. The latter include the alpha tropomyosin gene (*TPM1*), telokin (*MLCK_v7*), and heavy caldesmon (*CALD*). VSMC-specific splice variants likely require specialized components of the spliceosome found only in the VSMC lineage. In sharp contrast to the transcriptional events underlying VSMC-specific gene expression, the nature of VSMC-specific splicing is only marginally understood[13]. In addition to contractile genes that encode for proteins involved in the unique contractile properties of VSMC, a variety of matrix-associated genes are expressed that provide the essential anchor points for VSMC to remain stationary in the vessel wall and respond to appropriately coordinated extrinsic signaling inputs[14]. Finally, very recent data from the microRNA (miR) revolution has revealed a uniquely expressed miR (miR-143-145) that, like some VSMC contractile genes, shows early embryonic expression in cardiac muscle only to become specific for adult VSMC[15]–[17]. miRs are transcribed from the genome in the same manner as protein-coding genes and, through a series of RNAse III-dependent cleavage events, these small non-coding RNA sequences fine tune the proteome through partial Watson-Crick base pairing across the processed mRNA (with some bias for the 3′ un-translated region) resulting in either the repression of translation or the direct destabilization of target mRNA sequences[18]. In VSMC, the miR-143/145 bicistronic gene is uniquely expressed in postnatal vascular SMC with little to no expression throughout the rest of the adult body[15]–[17]. Both miR-143 and miR-145 play auxiliary roles in defining VSMC through their ability to control expression of key transcription factors that, in turn, directly activate (or repress) VSMC gene expression[15]–[17],[19],[20].

A myriad of signaling pathways has been described that positively regulate the VSMC differentiated phenotype. One strong stimulus for VSMC gene expression is stretch due to the increasing pressure exerted by the heart on the vascular tree[21],[22]. Another well-defined signaling pathway for VSMC differentiation is via TGFβ1 acting through two receptors with serine-threonine kinase activity[23]. The canonical signaling pathway for TGFβ1 involves the activation of SMAD4 and its nuclear translocation in concert with other SMADs to directly bind SMAD response elements near VSMC promoters or cooperate through protein-protein interactions with other transcription factors to direct VSMC-specific gene expression[24],[25]. During development, PDGF-BB arising from endothelial cells directs VSMC or pericyte differentiation as evidenced by the hemorrhagic phenotype in PDGF-B or PDGF-β1 receptor knockout mice[26],[27]. A recent elegant study showed how sonic hedgehog signaling directs the differentiation of adventitial progenitor cells into VSMC[28]. In addition to these well described pathways leading to VSMC differentiation, there exists numerous other signal transduction pathways that contribute to the VSMC differentiated phenotype including, p38 MAPK[29], calcineurin/NFAT[30],[31], sphingosine 1/2 phosphate and its G protein-coupled receptors[32],[33], BMP4[34], Notch[35], thrombin[36], PKGI alpha[37], NOX4 and redox signaling[38],[39], and RhoA[40]. As will be discussed next, there is a common genomic code for the transcriptional regulation of most VSMC-specific genes where essentially all of the signaling pathways converge.

## TRANSCRIPTIONAL SWITCH FOR VSMC DIFFERENTIATION

Most signal transduction pathways converge upon the nucleus to direct cell-specific gene expression through the action of DNA-binding transcription factors and a litany of co-regulators that are recruited to discrete elements bound by the signal-responsive transcription factor. In VSMC, as in cardiac and skeletal muscle, the principal DNA-binding transcription factor involved in cellular differentiation is serum response factor (SRF)[41]. SRF binds to at least 1,216 permutations of a *cis* element known as the CArG box[42]. Functional CArG boxes are more often found in the immediate vicinity of the transcription factor start site probably because of SRF's ability to work with components of the general transcriptional machinery[43]. Recent computational and wet-lab screening assays have greatly expanded the co-called CArGome to include, not only contractile genes, but cytoskeletal, signaling, and transcription factor genes as well[44]–[48]. All total, there are 217 validated CArG boxes in the genome with more than 300 awaiting wet-lab confirmation. Ironically, SRF-binding CArG boxes are also found in an array of growth-related genes[41],[49]. Indeed, the first SRF target gene described was *FOS*, which is known to be associated with the growth response of many cell types including VSMC[50]–[52]. A major conundrum in the field therefore was how a widely expressed transcription factor could “toggle” between distinct programs of gene expression, namely growth and differentiation. As described next, the discovery of a key SRF cofactor has revolutionized our understanding of the molecular key to the VSMC-specific program of differentiation.

The paradigm for differentiation of a multi-potential cell to one of more definitive nature carrying out specific functions to maintain homeostasis was established with MyoD in skeletal muscle. These seminal findings, first described by Andrew Lassar in the late Harold Weintraub's lab[53]–[55], demonstrated the importance of a single transcription factor (MyoD) in establishing and maintaining the skeletal muscle lineage. Subsequent studies across organ systems have exploited the beauty of this system to make inroads into the transcriptional basis for other cell types. In VSMC, this journey has been a long one beginning on the heels of the great MyoD discovery. Numerous labs used a variety of arduous, wet-lab methods to try and identify MyoD-like factors that could positively influence the VSMC differentiated phenotype. In the end, however, it took only the key strokes of a computer to find the elusive factor we now know as myocardin (MYOCD).

Dazhi Wang, in the laboratory of Eric Olson, sifted through EST databases for genes uniquely expressed in cardiac muscle and found, among many, a gene initially thought to be restricted only to cardiac muscle, hence its designation as *MYOCD*[56]. Remarkably, MYOCD makes physical contacts with SRF bound to CArG elements to increase gene transcription by several orders of magnitude[56]. Even more fascinating is the discrimination MYOCD makes between SRF-bound CArG elements in growth-related genes (*e.g., FOS* and *EGR1*) versus cardiac muscle genes; MYOCD does not effect growth-related gene expression. A subsequent study extended MYOCD's expression to cultured VSMC and adult aortic tissue and, predictably, this SRF cofactor greatly enhanced VSMC-specific promoter activity where CArG elements reside[57]. More importantly, using the classic MyoD conversion assay, which showed MyoD's ability to convert other cell types to skeletal muscle, overexpression of MYOCD in a non-VSMC cell type activated endogenous expression of CArG-containing VSMC-specific genes[57]. Indeed, MYOCD has been suggested to be a MyoD-like master regulator for VSMC-specific gene expression[58]. Moreover, MYOCD, like MyoD in skeletal muscle, is sufficient to direct structural and physiological attributes of VSMC[59]. A notable exception to the MyoD paradigm is MYOCD's apparent lack of intrinsic DNA binding to a canonical *cis* element, though the atomic structure of MYOCD bound to SRF over a CArG element has yet to be solved. Since the first description of MYOCD as a molecular switch for the VSMC differentiated phenotype[57], numerous other labs have confirmed and extended the finding that MYOCD directs endogenous VSMC-specific gene expression[58],[60]–[62]. Two additional MYOCD paralogs have been cloned and characterized called MRTFA and MRTFB, with the former exhibiting virtually identical activity to MYOCD[63]. Further evidence for MYOCD's role as an important regulator for VSMC differentiation is offered through gene inactivation studies that show defective VSMC-specific gene expression as well as a synthetic ultrastructural phenotype with much lower content of contractile myofilaments resulting in premature death[64],[65]. Similarly, SRF inactivation in VSMC results in embryonic lethality due to a reduction in VSMC contractile genes and altered cyto-contractile elements, likely because of MYOCD's inability to be anchored over CArG-containing VSMC-specific genes[66]. Taken together, the results from many labs have firmly demonstrated the importance of MYOCD (and SRF) for the appropriate expression of VSMC-specific genes and, by extension, the establishment of a functionally differentiated VSMC.

For more than 40 years, VSMC biologists have struggled to understand the basis for so-called VSMC phenotypic modulation. This process was initially described, inappropriately, as “de-differentiation” when VSMC were cultured *in vitro* and shown to exhibit accelerated growth with the loss of contractile properties[67]. Subsequent studies showed similar phenomena *in vivo* when blood vessels were subject to mechanical or dietary injury[68],[69]. With the identification of MYOCD as a master switch for VSMC-specific gene expression, it was clear from the start that VSMC phenotypic modulation was likely a manifestation of lowered MYOCD expression[57]. Indeed, in vivo arterial injury studies have shown repeatedly that MYOCD mRNA expression is reduced following balloon angioplasty or endothelial cell denudation[70],[71]. The molecular basis for MYOCD down-regulation, whether *in vitro* or *in vivo* following arterial insult, is not clear since the promoter to MYOCD is very complex. Recently, however, evidence has emerged for an important role of miR-145 in MYOCD regulation. Just as MYOCD is down-regulated under conditions favoring VSMC phenotypic modulation, miR-145 expression levels are greatly reduced after mechanical injury or diet-induced atherosclerosis[15],[19]. Increasing levels of miR-145 in the injured vessel wall appears to reconstitute normal expression of VSMC contractile genes[15],[19]. Moreover, MYOCD itself is elevated with miR-145 over-expression[15]. The latter result was surprising since miRs are generally thought to act as silencers. However, it appears that miR-145 augments MYOCD through its repression of two targets (KLF4 and KLF5)[15],[17],[19],[72] that themselves appear to repress MYOCD[19],[73]. Thus, VSMC phenotypic modulation is finally being understood in molecular terms through complex circuitry involving transcription factors and miRs that exert post-transcriptional control, either directly or indirectly, over key transcription factors. It will be of cardinal importance to determine whether VSMC phenotypic modulation can be thwarted in vivo with small molecules that stabilize the program of MYOCD/miR-145 expression.

Although MYOCD is without question, the critical factor for the establishment and maintenance of VSMC differentiation, there are additional transcription factors that play some role in this process as well. For example, GATA6 was shown to be down-regulated in the injured vessel wall concomitant with reduced contractile genes. Adenoviral-mediated gene transfer of GATA6 to the injured vessel wall restored contractile gene expression and reduced vascular neointimal formation[74]. Interestingly, GATA6 can displace MYOCD and thus reduce *MLCK_v7* (telokin) promoter activity and expression (which is low in VSMC) or synergize with MYOCD and activate *MYH11* promoter activity. In addition, GATA6 turns on other VSMC contractile genes[75]. More recently, statins were shown to up-regulate GATA6 binding activity to the *MYH11* promoter region and increase endogenous expression of some VSMC-specific contractile genes[76]. It will be of interest to assess the effects of statins on MYOCD expression or activity. C-MYB, which is most often linked to hematopoietic cell differentiation, has recently been implicated in VSMC differentiation as well. Embryonic stem cells lacking c-MYB were incapable of differentiating into contractile VSMC[77]. As might be expected, *MYOCD* expression levels were also compromised suggesting that c-MYB either directly or indirectly activates the *MYOCD* gene. Finally, a very recent study has shown that the NRF3 transcription factor, involved with redox control in a cell, positively enforces the VSMC contractile phenotype, in part, through the elevated binding of SRF and MYOCD to CArG elements as well as the up-regulation of MYOCD itself[78]. It is not clear at this time how NRF3 increases MYOCD expression. Collectively, these few examples highlight the fact that the VSMC differentiated phenotype, while determined mainly by levels of MYOCD, can be influenced by additional transcription factors as well.

## RETINOIDS AND VSMC PHENOTYPE

Even before MYOCD was discovered, labs world-wide were exploring ways to prevent VSMC phenotypic modulation, and by extension vascular disease, in an array of vascular injury models. One such foray involved the study of retinoids, which are natural and synthetic derivatives of vitamin A that act as ligands for nuclear transcription factors[79]. The motivation for studying retinoids came with the realization that the pathogenesis of arterial disease resemblance that of cancer[80], where retinoids were being evaluated as potential therapeutic drugs. Indeed, several retinoids, most notably all trans retinoic acid (ATRA; vesanoid), have shown variable efficacy in the treatment of several human cancers[81]. Application of ATRA or other synthetic retinoids (*e.g.*, Am80) to animals subjected to balloon angioplasty or an atherosclerotic regimen showed reductions in vascular occlusive disease[82]–[85]. Moreover, evidence exists showing a preservation of VSMC differentiation as revealed by the expression of VSMC-specific contractile genes/proteins[84]–[86]. At this time, there has been no study showing effects of retinoids on MYOCD expression or activity; however, given the positive influence of ATRA on VSMC marker expression, one might hypothesize that ATRA would augment MYOCD expression/activity. Certainly, studies should be performed addressing this question.

Retinoids exert their biological actions primarily through the activation of nuclear receptors that upon ligand activation, direct changes in gene expression. There are 6 retinoid receptors, three retinoic acid receptors (RAR) that mainly bind ATRA and three retinoid X receptors (RXR) that mainly bind the 9-cis stereoisomer of ATRA[87]. Cultured VSMC express all retinoid receptors except RXR gamma and respond to both ATRA and 9cis RA by exhibiting growth inhibition[88]. Both *in vitro* and *in vivo* studies of retinoids in the vessel wall prompted a screen for retinoid-response genes. Using a modified subtractive hybridization assay wherein cultured VSMC stimulated with or without ATRA, 14 novel retinoid-response genes were identified that showed either immediate early responses to ATRA that did not require *de novo* protein synthesis or delayed responses requiring new protein synthesis for induction[89]. Many genes were down-regulated by ATRA but none were followed up for further study. A brief summary of some of the more relevant retinoid-response target genes is provided next.

### Tissue transglutaminase

There are at least four transglutaminase genes whose encoded proteins play critical roles in the cross-linking of proteins[90]. Tissue transglutaminase (*TGM2*) exhibited very robust and early activation with ATRA and other stereoisomers of ATRA[91]. Moreover, the protein product was elevated as was the ability of TGM2 to cross-link known substrates. Importantly, TGM2-mediated programmed cell death in cultured VSMC suggesting retinoid-mediated growth suppression *in vivo* may involve an element of apoptosis[91]. Indeed, expression of TGM2 mRNA could be demonstrated in the neointima of balloon angioplastied carotid arteries[89].

### Alpha 8 integrin

The superfamily of integrin genes are involved with diverse biological properties such as growth, differentiation, and outside-in signaling events. Application of ATRA to cultured VSMC resulted in the delayed induction of alpha 8 integrin (*ITGA8*)[89]. Interestingly, ITGA8 protein expression is highly specific for VSMC[92] making this particular integrin subunit part of the molecular signature of VSMC (***Table 1***). Analysis of the 5′ promoter region reveals a conserved CArG box; however studies to date have failed to demonstrate this gene as an SRF target and mRNA levels do not appreciably change upon forced expression of MYOCD (unpublished). On the other hand, several other integrin genes are direct targets of SRF including *ITGA1*, *ITGA5*, and *ITGB1*[48],[93],[94]. Balloon injury to the vessel wall appears to up-regulate ITGA8[95], but rather than acting as a pro-proliferative or pro-migratory mediator, ITGA8 seems to block VSMC proliferation and migration[96],[97]. Moreover, ITGA8 can promote the expression of VSMC differentiation markers, possibly through the stimulation of actin filament polymerization and the nuclear translocation of MRTFA that together with SRF directs VSMC contractile gene expression[98]. Thus, retinoid-induced ITGA8 expression could confer, in part, the beneficial effects of retinoids seen in vivo following balloon injury, that is reduced proliferation and the promotion of a more differentiated VSMC phenotype.

### A-Kinase Anchoring Protein 12

In order for generic signaling to confer varied responses across each of the some 250 cell types, cells must compartmentalize signaling in a manner that best befits the cell's homeostatic balance. One manner in which cells do this is through the action of A-Kinase Anchoring Proteins (AKAPs) that can bind both effector protein (*e.g.*, a kinase) and downstream substrates of the effector protein[99]. In the screen for retinoid-response genes, the AKAP12 (aka SSeCKS) gene was induced within only a few hours of stimulation. Thus, AKAP12 is an immediate early retinoid-response gene[89]. AKAP12 has tumor suppressor properties and is indeed one of the only tumor suppressors known to be induced by retinoids. Early studies showed how AKAP12 could inhibit the growth and migration of cells, most notably cancer cells[100]. Analysis of the AKAP12 locus has revealed a complex organization with at least three independent transcription units each under control of its own promoter residing within a >100 kb gene locus[101]. Interestingly, the AKAP12 alpha gene is an atypical SRF target gene because while its paired CArG boxes bind SRF and are required for SRF-dependent promoter activity, they appear to be unresponsive to the two main signaling arms leading to SRF-dependent gene expression, namely MYOCD and the MAPK-ELK1 pathway[102]. Current work is focused on the knockout of AKAP12 and the retinoid responsive elements that must exist in one of the internal promoters.

### Vascular Cell Adhesion Molecule

Part of the VSMC program of differentiation involves the expression of various adhesion proteins required for the establishment of a sessile, non-motile state in the normal vessel wall. Although vascular cell adhesion molecule 1 (VCAM-1) is largely known for its important role in endothelial cells during fatty streak formation in early atherogenesis, this marker of inflammation is also known to be expressed in VSMC. In fact, the initial knockout of VCAM-1 exhibited a defect in VSMC differentiation[103]. A subsequent report indicated that the concurrent induction of VCAM-1 with the gold standard marker of VSMC differentiation, *MYH11*[104], occurred independently of NF-κB[105]. Thus, VCAM-1 appears to have distinct functions in VSMC related to the differentiated phenotype and the inflammatory response to such agonists as TNFα[105],[106]. ATRA was shown to consistently up-regulate *VCAM-1* mRNA in cultured VSMC[89]. It is thus tempting to speculate that expression of this adhesion molecule with ATRA contributes to the known effects of retinoids on the differentiated phenotype of VSMC; it will be imperative to elucidate the mechanisms through which VCAM-1 confers a VSMC differentiated state.

### D9

One of the “genes” found to be induced with ATRA in VSMC goes by the provisional name of *D9*. Initial Northern blotting studies revealed that *D9* is a small transcript (less than 1 kb) suggesting that it could represent a novel microRNA. Attempts to clone other orthologs of *D9* from other species have been unsuccessful and screening of a rat genomic library failed to uncover a genomic clone. Remarkably, sequence analysis of *D9* has failed to reveal any homology to anything in all of the annotated databases. At this period of time, we are completely at a loss as to whether *D9* represents an unknown protein coding gene, a non-protein coding RNA gene or something vestigial to an infection of the rat genome from which we derived the initial VSMC for the retinoid-response gene screen.

### Retinoid-Inducible Serine Carboxypeptidase (*RISC*)

One of the retinoid-response genes originally cloned was a novel gene we called *RISC* due to its amino acid homology to a large family of plant serine carboxypeptidases bearing a classic catalytic triad comprising the amino acids serine, aspartic acid, and histidine[107]. This name was quickly changed to serine carboxypeptidase 1 (*SCPEP1*) because of the emerging field of *RISC* biology and more importantly, the subsequent finding that *SCPEP1* is not induced by retinoids. Nevertheless, *SCPEP1* is expressed in VSMC and across multiple tissues as shown by antibody studies[108]. There are at least two variants of SCPEP1 resulting from an apparent proteolytic cleavage event near the carboxy terminal end of the protein[108],[109]. SCPEP1 is a lysosomal protein and its cleavage appears to occur in the lysosome since treatment of VSMC with chloroquine blocks cleavage (unpublished). Despite extensive surveying and testing, there are no known substrates for SCPEP1 making this protein an “orphan protease”. Genetic inactivation of *SCPEP1* does not show an overt phenotype as mice survive and breed without any histological evidence of pathology[109],[110]. However, upon ligation injury of the carotid artery, *SCPEP1* null mice show reduced neointimal load suggesting that SCPEP1 directs VSMC migration and proliferation. Indeed, adenoviral delivery of SCPEP1 to VSMC causes accelerated growth and migration in a catalytic triad-dependent manner[110]. Interestingly, SCPEP1 is secreted from cells in a non cleaved manner though it is not clear as yet whether extracellular SCPEP1 exhibits biological activity. There is much work to be done on SCPEP1 including the elucidation of its substrates and precise functions in the vessel wall as well as its molecular control in gene expression.

Several other retinoid-response genes in VSMC exist that await further study. These include endolyn, ceruloplasmin, importin alpha, cathepsin-L, and an unusual transcription factor called SALF that results from the fusion of two co-transcribed genes[89]. One of the future goals should be to ascertain whether these and other retinoid-response genes harbor retinoic acid response elements that bind the ligand-activated nuclear retinoid receptors. Another goal should be to find out if, like AKAP12 alpha[102], any of the genes show SRF-dependency for expression and, if so, whether SRF incorporates MYOCD or some other cofactor to direct gene expression. Finally, whether retinoids such as Am80 will make their way into the clinic for the treatment of arterial disease in conjunction with standard therapies (*e.g*., eluting stents) is unclear at the moment. One would think that given the years of clinical experience with retinoids for the treatment of cancer and dermatopathologies, that an application in the setting of certain vascular diseases such as recalcitrant transplant arteriopathies would be warranted.

## PERSPECTIVES AND FUTURE DIRECTIONS

**Fig. 1:**
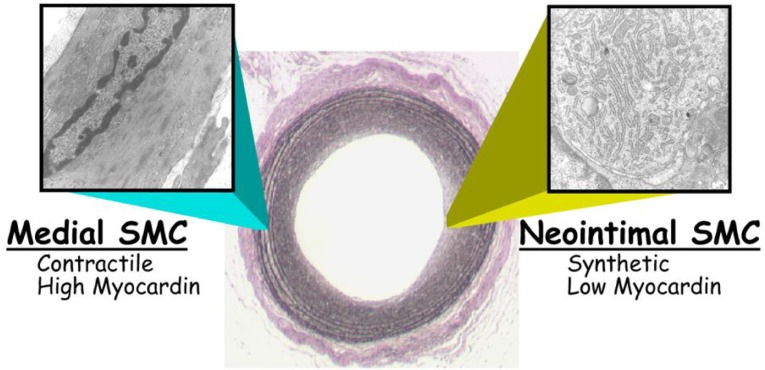
Phenotypic modulation of arterial smooth muscle cells. Shown is a micrograph from a rat carotid artery 14 days following balloon de-endothelialization with transmission electron micrographs taken from either the tunica media prior to injury (left) or within the neointima at 14 days (right). Note the marked ultrastructural changes accompanying the neointimal lesion at right where myocardin levels are considered to be lower, leading to loss in SRF-dependent contractile filaments and the emergence of the classic synthetic phenotype with a rich quantity of rough endoplasmic reticulum. Myocardin levels are thus seen as a critical determinant of either the contractile state (left, with high myocardin) or the less differentiated, synthetic phenotype (right, with low myocardin). See text for details.

A series of scientific revolutions has occurred in only the last 57 years since the elucidation of the structure of DNA[111] that heralded the age of molecular biology and its subsequent confluence with the genomics and bioinformatics revolutions. We are now poised to gain the most fundamental insights into what it is, biologically, to be human and, by extension, how normal human life processes are subverted in disease states such as cancer, cardiovascular disease, and neurodegenerative disorders such as Alzheimer's disease. VSMC represent only one of some 250 distinct cell types that exist in humans to establish normal life processes. The existence of an SRF-MYOCD molecular switch for the VSMC differentiation program allows for an unprecedented molecular view into the inner workings of these cells during development and in postnatal disease states such as occurs following iatrogenic injuries (***Fig. 1***). Future studies should exploit mouse models of knocking each component of the switch out at discrete times during embryonic or postnatal development. Further, it will be necessary to fully disclose the SRF-MYOCD program of gene expression in VSMC using, for example, ChIP-sequence in both wildtype and knockout conditions. How this switch interfaces with other transcriptional (and post-transcriptional) events is also an important goal as is the definition and rules of signaling that govern VSMC differentiation. Atomic structure studies would also be of great utility in the design of small molecules that could either impede interactions between SRF and MYOCD or promote expression or activity of this switch so as to maintain a normal VSMC phenotype in the face of disease. The information acquired thus far, coupled to the array of tools we have in hand, should be enticing for the next generation of scientists interested in further expanding our knowledge base of the differentiated VSMC phenotype.
